# Surgical management of petrous apex lesions: a descriptive analysis of outcomes by anatomical location for the Kawase, retrosigmoid, and pterional approaches

**DOI:** 10.3389/fneur.2026.1758336

**Published:** 2026-02-26

**Authors:** Tianyang Wu, Hao Lang, Meiqi Wu, Xialin Zheng, Shan Xie, Longjie Cai, Dongqi Shao, Yu Li, Zhiquan Jiang

**Affiliations:** Department of Neurosurgery, The First Affiliated Hospital of Bengbu Medical University, Bengbu, China

**Keywords:** anatomical location, Kawase approach, neuroendoscopy, petrous apex, pterional approach, retrosigmoid approach, tumor

## Abstract

This study is a descriptive analysis that systematically delineates the perioperative outcome profiles of Kawase, endoscope-assisted retrosigmoid, and pterional approaches for resecting petrous apex lesions within the clinical decision-making framework of “anatomical location first.” A retrospective series of 27 patients was included. Based on the core anatomical location of the lesion, surgery was performed via the Kawase approach (anteromedial region, *n* = 14), the endoscope-assisted retrosigmoid approach (posterior region, *n* = 7), or the pterional approach (superoanterior region, *n* = 6). The results demonstrate that surgical approaches corresponding to different anatomical subregions exhibited characteristic outcome profiles. For anteromedial petrous apex lesions, the Kawase approach achieved a high gross total resection rate (100%), but was associated with longer operative time and a higher risk of postoperative intracranial infection. For posterior lesions, the endoscope-assisted retrosigmoid approach provided excellent exposure with minimal tissue trauma and a relatively balanced complication spectrum. For superoanterior lesions, the pterional approach, while allowing direct access, was associated with higher rates of postoperative cranial nerve dysfunction (trigeminal nerve injury 50%, facial nerve palsy 67%) and speech impairment (50%). In a subgroup analysis focusing on the predominant pathology (meningioma, *n* = 20), these outcome differences linked to specific anatomical location-approach pairings persisted. The findings indicate that surgical outcomes for petrous apex lesions are closely associated with the approach dictated by their anatomical location, presenting a predictable characteristic profile. Therefore, clinical decision-making should prioritize the precise anatomical location of the lesion when selecting the surgical approach, and fully acknowledge the inherent perioperative risk profile specific to each “anatomical region–surgical approach” pairing. The integration of ancillary techniques such as neuroendoscopy with classic approaches holds promise for further optimizing outcomes in complex cases. This study is a single-center, retrospective, descriptive analysis with a limited sample size; its conclusions require validation by prospective, large-sample studies.

## 1 Introduction

The petrous apex is one of the most challenging regions in neurosurgery, surrounded by numerous critical nerves and blood vessels. The choice of surgical approach for petrous apex lesions significantly influences treatment outcomes. This study systematically delineates the perioperative outcome profiles of three surgical approaches for petrous apex lesions, based on the clinical decision-making framework of ‘anatomical location first’.

The petrous apex is a pyramid-shaped structure formed by the medial portion of the temporal bone. It is bounded laterally by the inner ear structures, medially by the petro-occipital fissure, anteriorly by the petrosphenoidal fissure and the internal carotid artery, and posteriorly by the posterior cranial fossa. Its superior surface is related to the middle cranial fossa, Meckel’s cave, and the internal carotid artery, while its inferior surface is related to the jugular bulb and inferior petrosal sinus. The petrous apex is divided by the internal auditory canal into a larger anterior part and a smaller posterior part. The anterior part extends anterior to the cochlea and is the most common site for pathology. The posterior part lies between the internal auditory canal and the semicircular canals and consists predominantly of dense otic capsule bone. The anterior compartment of the petrous apex contains the horizontal segment of the petrous carotid artery and the foramen lacerum. The abducens nerve courses along its medial surface, while the trigeminal nerve passes immediately superomedial to it. The internal auditory canal, located in the mid-portion of the petrous apex, transmits the vestibulocochlear and facial nerves (1, 2). The anterior part of the petrous apex is largely composed of bone marrow, which undergoes pneumatization. Air cells extend into the petrous apex through various tracts, which communicate directly with the mastoid or middle ear cavities, providing direct pathways for the development of pathologies in this region (3).

Petrous apex lesions encompass inflammatory lesions, neoplastic lesions, and other conditions. Inflammatory lesions include cholesterol granulomas and cholesteatomas. Neoplastic lesions comprise meningiomas, schwannomas, chondrosarcomas, and metastatic tumors. Other pathologies include craniocervical junction aneurysms and pneumatization-related disorders. The most common clinical manifestation is hearing impairment. Other potential symptoms include vestibular dysfunction (manifesting as dizziness, nausea, and vomiting), headache, tinnitus, hemifacial spasm, diplopia, facial paralysis, and even otorrhea (4, 5).

Due to its deep-seated location and the complex neurovascular structures it contains, surgical management of the petrous apex is particularly challenging. Therefore, selecting an appropriate surgical approach is crucial for achieving a high lesion resection rate and minimizing complications.

The Kawase approach, also known as the anterior petrosectomy or subtemporal transpetrosal approach, provides extensive surgical exposure and working space. This includes access to the petrous segment of the internal carotid artery, the trigeminal nerve, the internal auditory canal, and an extradural corridor between the facial-vestibulocochlear nerve complex (CN VII-VIII), and offers improved visualization of the posterior fossa. It is utilized for treating lesions such as upper and mid-clival tumors, epidermoid cysts, and aneurysms in the ambient cistern region (6, 7). Despite its advantages, the Kawase approach presents challenges, including difficulties in nerve preservation and a steep technical learning curve for surgeons. The retrosigmoid approach is a common technique for neurosurgeons, providing excellent exposure of the cerebellopontine angle, the ventrolateral brainstem, and cranial nerves V through XII. When combined with neuroendoscopy, it can enhance visualization of the petrous apex. However, its trajectory to the petrous apex is relatively long, and it is not suitable for all petrous apex pathologies (8). Similarly, the pterional approach is a frequently used technique that offers good exposure of the superior petrous apex, the middle fossa floor, and the sellar region. Its limitations also include a long working distance to the petrous apex, potential challenges in nerve preservation, and limited applicability to certain petrous apex lesions (9).

However, in clinical practice, conducting prospective controlled studies directly comparing different surgical approaches is challenging because approach selection is primarily determined by the specific anatomical location of the lesion. Therefore, under this anatomy-driven framework, systematically delineating the perioperative outcomes associated with each standard approach is of significant importance for guiding preoperative decision-making, optimizing surgical planning, and informing prognosis.

Consequently, this study retrospectively analyzed 27 patients with petrous apex lesions, all operated on by the same surgical team. Adhering to the principle of prioritizing anatomical lesion location, we systematically described the technical features, complication profiles, and tumor control outcomes associated with the Kawase, endoscope-assisted retrosigmoid, and pterional approaches. This provides a valuable reference for preoperative counseling and surgical planning.

## 2 Patients and methods

### 2.1 Patients’ summary

This retrospective study included patients with intracranial petrous apex lesions who underwent surgical treatment in the Department of Neurosurgery at the First Affiliated Hospital of Bengbu Medical University between January 2020 and August 2025. All patients were diagnosed with petrous apex lesions via preoperative magnetic resonance imaging (MRI). The cohort comprised 10 male and 17 female patients. Surgical approaches included the Kawase approach (14 patients), the retrosigmoid approach combined with neuroendoscopy (7 patients), and the pterional approach (6 patients). Postoperative pathological examination revealed meningioma in 20 patients, trigeminal schwannoma in 3 patients, cyst in 2 patients, chordoma in 1 patient, and cholesteatoma in 1 patient.

Inclusion criteria:

1. All patients were diagnosed with space-occupying lesions in the petrous apex via MRI and other relevant examinations.
2. All patients underwent surgical treatment at our institution.
3. Patients had no other severe, relevant organic comorbidities.
4. Informed consent was obtained from all patients for participation in this study.

Exclusion criteria:

1. Presence of other severe, relevant organic comorbidities.
2. History of psychiatric disorders impairing effective communication with physicians.
3. History of other malignant tumors.
4. Concurrent active infectious diseases.
5. Unwillingness to provide informed consent for the study.

Prior to surgery, a team of neurosurgical specialists evaluated the tumor location to determine the most appropriate surgical approach. The team then comprehensively explained the specific surgical plan to the patient and their family, including the advantages, risks, and prognosis associated with the chosen approach. This ensured the patient fully understood the treatment strategy and provided informed consent.

### 2.2 Criteria for selecting the surgical approach

The surgical approach was selected through standardized multidisciplinary discussions involving senior neurosurgeons, neuroradiologists, and neuropathologists. The final decision was primarily based on the following imaging and clinical criteria:

#### 2.2.1 Lesion location (primary determinant)

The Kawase approach is suitable for lesions located in the anteromedial petrous apex, particularly for those extending into the middle and/or posterior cranial fossae.

The retrosigmoid approach is suitable for lesions located in the posterior petrous apex or those extending into the cerebellopontine angle.

The pterional approach is suitable for lesions in the superoanterior petrous apex with extension into the parasellar region or cavernous sinus.

#### 2.2.2 Critical structures involved

The involvement of key peri-lesional structures—specifically (a) cavernous sinus, (b) Meckel’s cave, (c) transtentorial extension, and (d) clivus—was systematically recorded. The extent of involvement was then evaluated to inform the selection of the surgical approach.

#### 2.2.3 Relationship between tumor size and neurovascular structures

Preoperative MRI and CT angiography were used to evaluate the anatomical relationships of the lesion with the internal carotid artery, basilar artery, cranial nerves (CN III–XII, i.e., oculomotor nerve to hypoglossal nerve), and brainstem. The surgical approach was chosen with the goal of maximizing exposure while minimizing retraction and manipulation of these critical structures.

#### 2.2.4 Imaging characteristics of pathological types

Although definitive pathological diagnosis is not obtained until after surgery, preoperative imaging characteristics can provide additional insights into estimated tumor consistency and aggressiveness, thereby informing the selection of the surgical approach.

#### 2.2.5 Patient-specific factors

Patient-specific factors, including prior intracranial neurological deficits and the overall clinical condition, were taken into careful consideration.

All cases were discussed collectively, and the final surgical approach was determined and documented preoperatively. This standardized process ensured consistency and reproducibility across all surgical plans.

### 2.3 Surgical team

All surgical procedures in this study were performed by the same neurosurgical team at our institution, led by a senior neurosurgeon with extensive experience in skull base surgery. For each case, the surgical approach was determined through multidisciplinary discussion within this team. The primary surgeon participated in procedures utilizing all three approaches. This standardized protocol was implemented to minimize operator bias and to ensure that the observed outcomes primarily reflected the inherent characteristics of the surgical approaches themselves, rather than inter-surgeon variability.

### 2.4 Statistical methods

Data analysis was performed using SPSS Statistics version 31.0. Continuous variables conforming to a normal distribution are presented as mean ± standard deviation (x̄ ± SD). Due to the small overall sample size (*n* < 30), the Kruskal-Wallis test (a non-parametric test) was used for continuous variables, and Fisher–Freeman–Halton’s exact test was used for categorical variables. A *p*-value of less than 0.05 was considered statistically significant.

### 2.5 Surgical procedure

#### 2.5.1 Kawase approach

**Figure 1:**
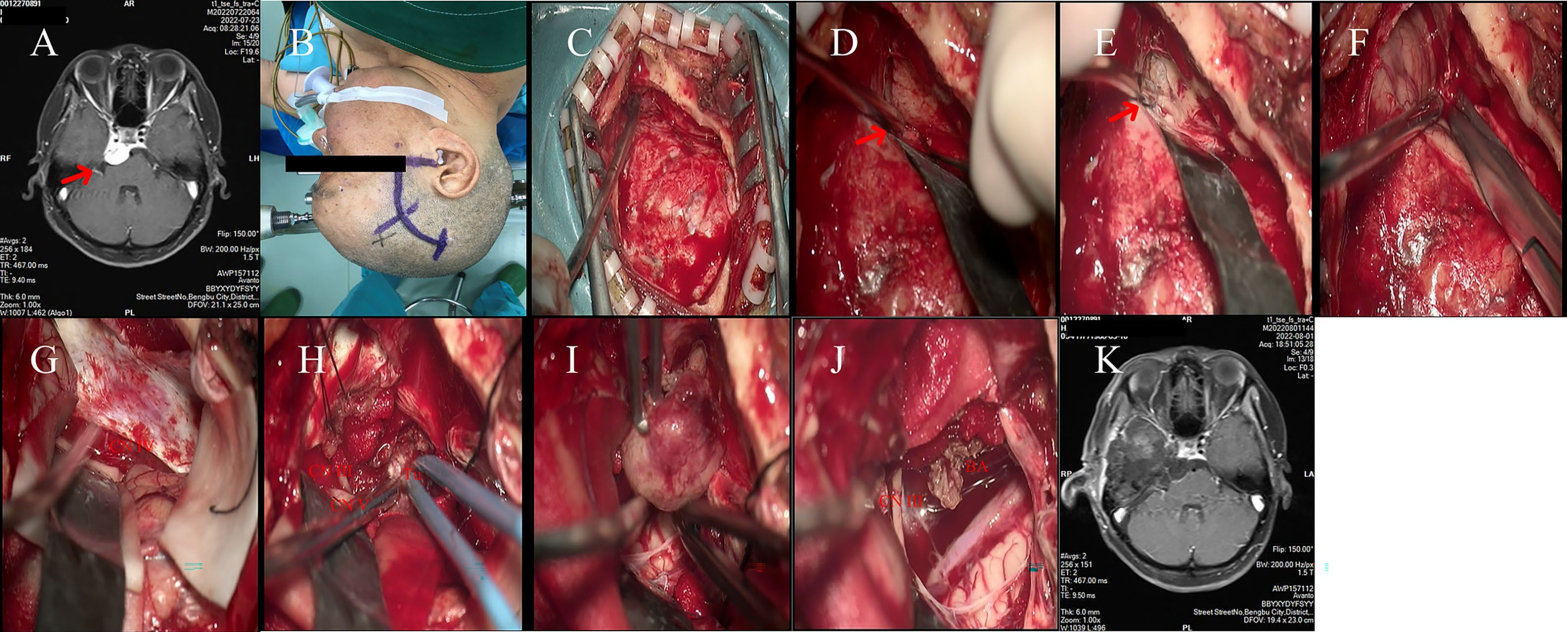
**(A)** Preoperative MRI indicates a mass located in the right petrous apex of the patient. **(B)** The right Kawase approach was selected. **(C)** The extent of the bone window. **(D)** Exposure of the petrous apex. **(E)** The petrous apex after drilling with a burr. **(F)** The meninges are incised with meningeal scissors, and cerebrospinal fluid is slowly released. **(G)** The trochlear nerve and the tumor can be observed. **(H)** The tumor tissue, oculomotor nerve, and trigeminal nerve can be observed. **(I)** The tumor is being removed gradually. **(J)** After tumor removal, the basilar artery and oculomotor nerve can be observed. **(K)** Postoperative MRI shows complete resection of the tumor.

Following orotracheal intubation, general anesthesia was administered. After successful anesthesia induction, the patient was placed in a supine position with the head rotated laterally and secured using a DORO head clamp (Germany). An 8 cm curvilinear incision was marked within the preauricular hairline, extending from the root of the zygomatic arch to above the pinna (Figure 1B). Routine skin disinfection and draping were performed.

The scalp layers were incised along the marked line using a surgical blade. A retractor was placed for exposure (Figure 1C). A burr hole was created in the skull, and a bone flap was turned down level with the zygomatic arch using a craniotome. The anterior portion of the petrous bone was drilled away (Figures 1D,E). The dura mater was opened in a “Y” shaped incision. Under microscopic magnification (3x), cerebrospinal fluid was aspirated, exposing the arcuate eminence of the petrous bone (Figure 1F). Dissection proceeded deeper towards the tentorial hiatus.

Approximately 1 cm anterior to the arcuate eminence, the dura at the tentorial hiatus was incised, revealing the trochlear nerve and the petrous apex tumor (Figure 1G). The dural incision was extended laterally, exposing the trigeminal nerve, oculomotor nerve, and Meckel’s cave (Figure 1H). A portion of the dura mater lateral to these structures was removed. The tumor contents were aspirated, and a sample was sent for pathological examination (Figure 1I).

Following the evacuation of the contents, the basilar artery became visible. The surgical field was repeatedly irrigated, and portions of the tumor capsule were removed (Figure 1J). After thorough irrigation and confirmation of hemostasis, the surgical field was clean with no active bleeding observed.

The dura was closed primarily. A sutureless artificial dura graft was applied to repair the dural defect. The bone flap was repositioned and fixed with three titanium clamps. The scalp layers were closed in sequence.

#### 2.5.2 Retrosigmoid approach

**Figure 2:**
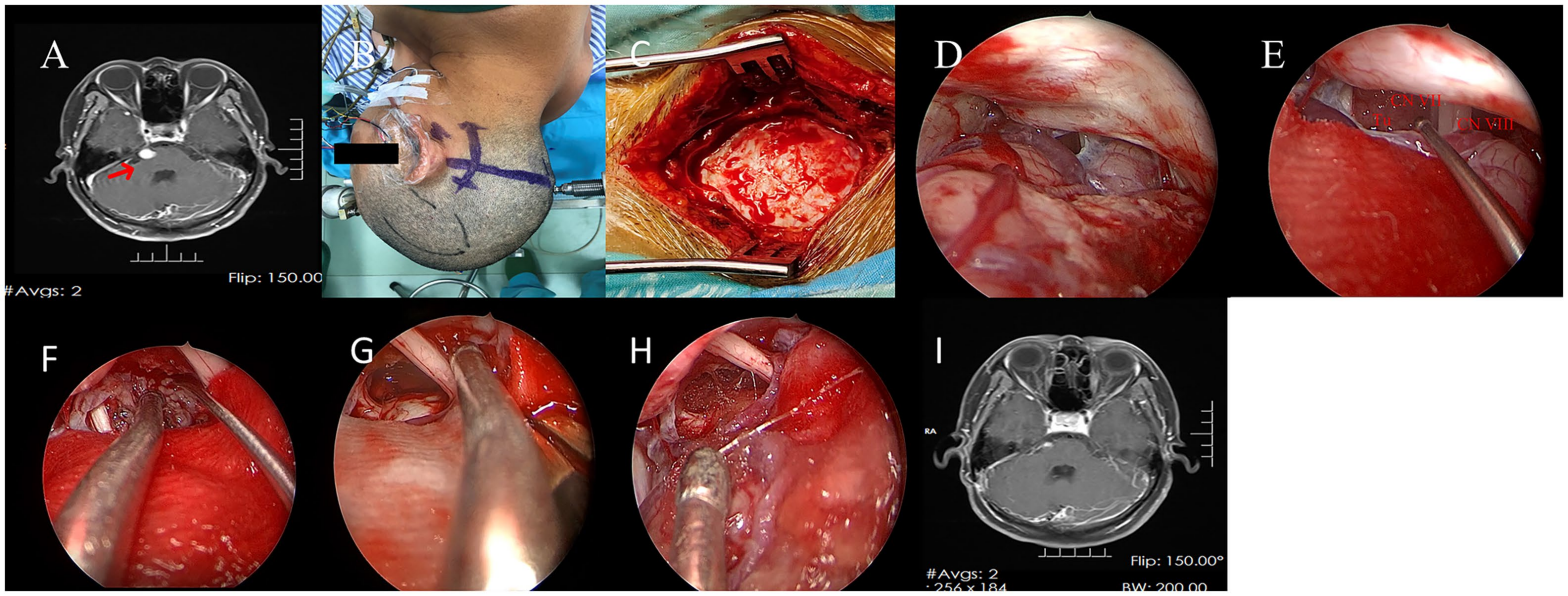
**(A)** Preoperative MRI revealed a mass in the patient’s right petrous apex. **(B)** The right retrosigmoid approach was selected. **(C)** The extent of the bone window. **(D)** Cerebrospinal fluid was slowly drained, achieving satisfactory brain relaxation. **(E)** Intraoperative exploration visualized the tumor, facial nerve, and vestibulocochlear nerve. **(F)** The tumor was gradually removed. **(G)** The surgical cavity after tumor resection. **(H)** Hemostasis was achieved using Surgicel and fluid gelatin, with no significant bleeding observed. **(I)** Postoperative MRI confirmed complete tumor resection.

Following orotracheal intubation, general anesthesia was administered. With the patient in a lateral position, the head was slightly flexed to facilitate exposure of the posterior mastoid region and secured using a DORO head clamp (Germany). A curvilinear incision was marked posterior to the mastoid, following the standard retrosigmoid approach (Figure 2B). Routine skin disinfection and draping were performed.

The skin, subcutaneous tissue, and suboccipital muscle layers were incised using a surgical blade until exposing the occipital bone. A retractor was used to laterally retract the suboccipital muscles. A burr hole was created, followed by craniotomy to elevate a free bone flap on the affected side, exposing the transverse and sigmoid sinuses (Figure 2C).

Under microscopic visualization, the dura was incised, and cerebrospinal fluid was released to achieve satisfactory brain relaxation (Figure 2D). The lateral aspect of the cerebellum on the affected side was explored, revealing the tumor located at the petrous apex (Figure 2E).

Neuroendoscopy was introduced, confirming the tumor was encapsulated and highly vascularized, with its base attached to the petroclival dura. The tumor base was first disconnected, followed by piecemeal resection of the lesion (Figure 2F). The facial nerve, vestibulocochlear nerve, lower cranial nerves (IX-XI), trigeminal nerve, basilar artery, and anterior inferior cerebellar artery were identified, carefully dissected, and preserved (Figure 2H).

Hemostasis within the tumor cavity was meticulously achieved. The dura was closed and repaired, the bone flap was repositioned and fixed, and the scalp layers were closed in sequence.

#### 2.5.3 Pterional approach

**Figure 3:**
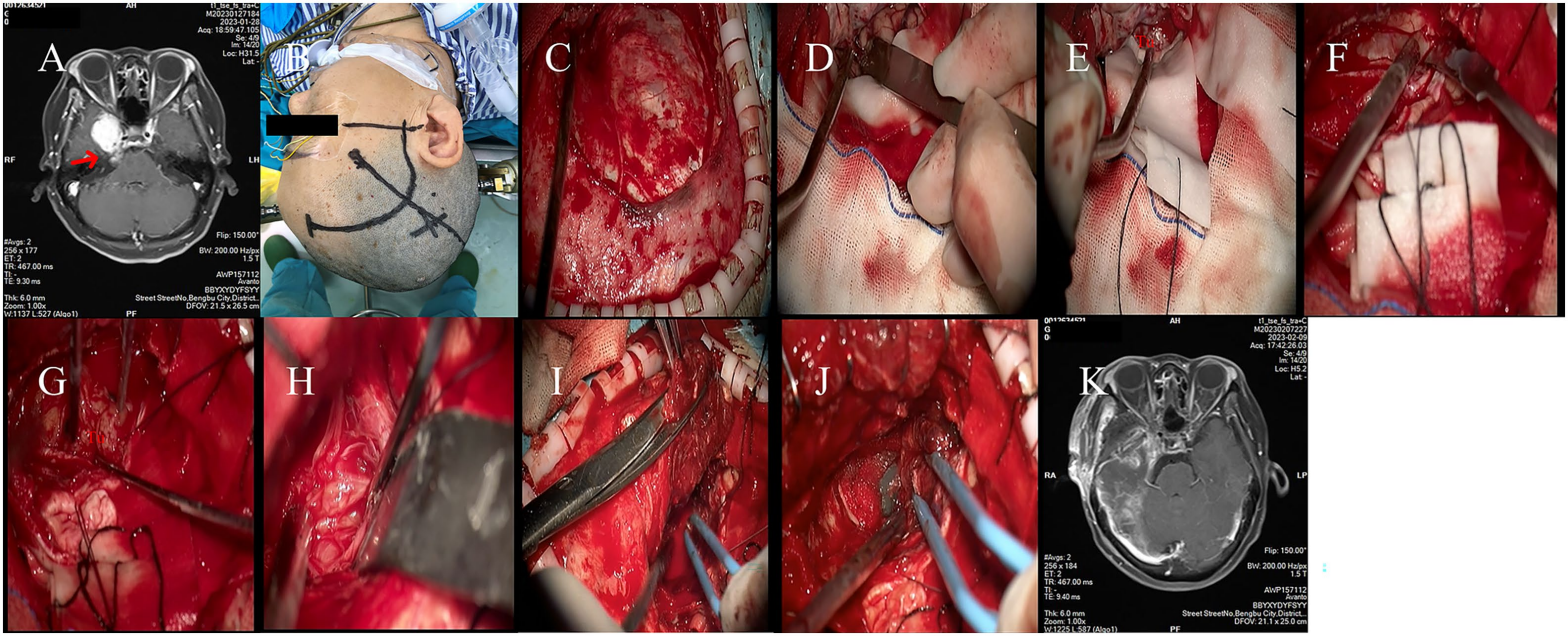
**(A)** Preoperative MRI revealed a large mass in the right petrous apex and parasellar region of the patient. **(B)** The right pterional approach was selected. **(C)** The extent of the bone window. **(D)** Cerebrospinal fluid was released from the lateral cisterns, achieving satisfactory brain relaxation. **(E)** After retracting the temporal lobe, the skull base tumor became visible. **(F)** Further exploration identified that the tumor base was located extradurally. **(G)** The tumor was resected in a piecemeal fashion. **(H)** Resection of the tumor portion extending into the posterior cranial fossa. **(I)** A segment of the temporal muscle was harvested. **(J)** Repair was performed using the temporal muscle. **(K)** Postoperative MRI confirmed complete tumor resection.

Following orotracheal intubation, general anesthesia was administered. After successful anesthesia induction, the patient was placed in a supine position with the head rotated laterally and secured using a DORO head clamp (Germany). A curvilinear incision was marked along the pterion on the ipsilateral side (Figure 3B). Routine skin disinfection and draping were performed.

Using a surgical blade, the scalp and subcutaneous tissues were incised sequentially. A skin flap was elevated beneath the galea aponeurotica, followed by dissection of the temporal muscle and periosteum. A burr hole was created, and a craniotome was used to turn a free bone flap (Figure 3C). The dura surrounding the bone window was tacked up, and fluid gelatin was applied epidurally to assist with hemostasis.

The inferior edge of the bone window extended to the middle cranial fossa floor (below the level of the zygomatic arch). The sphenoid ridge was flattened using a drill. Under microscopic visualization and with the use of an automatic brain retractor, the dura was opened, and cerebrospinal fluid was released to achieve satisfactory brain relaxation (Figure 3D).

The temporal lobe was gently elevated with the brain retractor, exposing the tumor located in the parasellar and petroclival regions (Figure 3E). The tumor was resected under microscopic guidance. Hemostasis was achieved with the assistance of Surgicel (Figure 3G). The dural defect was repaired using an artificial dura substitute.

The bone flap was repositioned and fixed. The wound was closed in layers and dressed.

### 2.6 Follow-up protocol

Follow-up data were collected by reviewing patients’ outpatient follow-up records, supplemented by telephone interviews when necessary. The follow-up period was calculated from the date of discharge to the date of the last available clinical or imaging assessment. As this was a retrospective study, not all patients were subject to a standardized, protocol-driven follow-up schedule. Consequently, the availability and timing of follow-up assessments were inconsistent across the cohort. The primary follow-up measures collected included.

#### 2.6.1 Radiological assessment

Evidence of tumor recurrence or progression on MRI.

#### 2.6.2 Neurological status

Assessment of intracranial nerve function (specifically the trigeminal and facial nerves), muscle strength, and speech.

#### 2.6.3 Complication-related outcomes

Assessment of recurrence of postoperative complications (e.g., CSF leak or intracranial infection). (All patients were discharged only after resolution of CSF leak and intracranial infection.)

### 2.7 Definition and assessment of complications

The major complications evaluated in this study were defined as follows.

#### 2.7.1 Intracranial infection

Diagnosis was established when at least two of the following clinical criteria were met postoperatively and confirmed jointly by a neurosurgeon and an infectious disease specialist.

1. Clinical manifestation: persistent fever (body temperature > 38.3 °C), persistent headache, neck stiffness, and even impaired consciousness, among other symptoms.
2. Laboratory criteria: Cerebrospinal fluid (CSF) analysis showing a leukocyte count > 100 cells/μL, and/or decreased glucose concentration (< 50% of the blood glucose level), and/or elevated protein level (> 0.45 g/L); or a positive CSF bacterial or fungal culture.
3. Radiological evidence: CT or MRI demonstrating new or progressive findings consistent with meningitis, cerebral edema, or brain abscess.

#### 2.7.2 Cerebrospinal fluid leak

A cerebrospinal fluid (CSF) leak was defined as the postoperative presence of either a subcutaneous fluid collection at the surgical site or continuous/intermittent drainage of clear fluid from the wound, nares, or ear canal, with biochemical confirmation of the fluid as CSF.

#### 2.7.3 Cranial nerve dysfunction

1. Facial nerve function: Facial nerve function was assessed using the House-Brackmann grading (HB) system, with a postoperative grade of III or higher defined as facial nerve dysfunction.
2. Trigeminal nerve function: Trigeminal nerve function was evaluated via standardized neurological examination, documenting patient-reported facial hypoesthesia, numbness, or pain, along with objective testing of light touch and pinprick sensation across all three trigeminal dermatomes. Trigeminal nerve injury was defined as any newly developed postoperative sensory abnormality or deficit.
3. Hearing function: Hearing was assessed by pure-tone audiometry. Hearing loss was defined as a pure-tone average (at 500, 1000, 2000, and 4,000 Hz) greater than 25 dB HL. (Preoperative hearing loss was recorded as an important baseline neurological status; however, because this was a retrospective study and hearing preservation was not a primary surgical goal in all cases, systematic and routine audiometric data (e.g., pure-tone audiometry) were not consistently collected.)

#### 2.7.4 Other functions

1. Motor function: Motor function was assessed using the Medical Research Council (MRC) scale for muscle strength grading. Motor weakness was defined as a muscle strength grade of 4 or lower.
2. Speech function: Speech impairment was defined as postoperative onset of dysarthria or aphasia, confirmed by bedside language assessment (including spontaneous speech, repetition, naming, and comprehension).
3. Consciousness status: Level of consciousness was assessed using the Glasgow Coma Scale (GCS). Consciousness disturbance was defined as a GCS score of less than 15 points.

#### 2.7.5 Extent of resection

1. Gross total resection (GTR): GTR was defined as the absence of any detectable tumor residue on MRI performed within 24 to 72 h postoperatively.
2. Near-total resection (NTR): NTR was defined as either a residual tumor volume of less than 5% of the original tumor volume, or only a thin layer of tumor capsule adherent to critical neurovascular structures, as demonstrated on MRI within 24 to 72 h postoperatively.

All imaging evaluations were independently performed by a senior neurosurgeon and a senior radiologist. Any discrepancies in their assessments were resolved through consensus discussion.

## 3 Results

### 3.1 Patient baseline characteristics

This study included 27 patients. Their detailed baseline characteristics, grouped according to the three surgical approaches, are presented (Table 1). In this study, the selection of surgical approach strictly adhered to the principle of “anatomical location first.” Consequently, as shown in Table 1, there were expected and systematic differences in the core anatomical localization of the tumors among the three patient groups. All patients who underwent the Kawase approach had lesions located in the anteromedial petrous apex (14/14, 100%), with frequent transtentorial extension (42.9%). All patients who underwent the retrosigmoid approach had lesions located in the posterior petrous apex (7/7, 100%). All patients who underwent the pterional approach had lesions located in the superoanterior petrous apex (6/6, 100%), with frequent cavernous sinus involvement (66.7%). Despite these clinically determined anatomical differences, the three groups did not differ significantly in terms of age, sex, tumor laterality, maximum tumor diameter, or preoperative key neurological functional status (e.g., hearing loss, facial numbness) (all *p* > 0.05). An identical anatomical distribution pattern was also observed in the largest pathological subgroup of meningioma patients (Table 2). This provides a relevant baseline for subsequent observation of outcome differences among the surgical approaches.

**Table 1:** Baseline characteristics of patients associated with the three surgical approaches.

Parameter	Kawase approach (*n* = 14)	Retrosigmoid approach (*n* = 7)	Pterional approach (*n* = 6)	*P*-value
Age (years)	54.13 ± 1.65	58.86 ± 0.71	58.20 ± 1.24	0.056
Gender				0.212
Male	3 (0.21)	4 (0.57)	3 (0.50)	
Female	11 (0.79)	3 (0.43)	3 (0.50)	
Tumor laterality				0.592
Left	9 (0.64)	3 (0.43)	4 (0.67)	
Right	5 (0.36)	4 (0.57)	2 (0.33)	
Maximum tumor diameter (cm)	2.94 ± 0.31	2.77 ± 0.55	2.98 ± 0.54	0.930
Meningioma	2.49 ± 0.30 (*n* = 8)	2.77 ± 0.55 (*n* = 7)	2.48 ± 0.25 (*n* = 5)	0.953
Other pathological types #	3.55 ± 0.53 (*n* = 6)	–	5.50 (*n* = 1)	–
Petrous apex subdivision				<0.001*
Anteromedial	14 (1.00)	0 (0.00)	0 (0.00)	
Posterior	0 (0.00)	7 (1.00)	0 (0.00)	
Superoanterior	0 (0.00)	0 (0.00)	6 (1.00)	
Extension				
Cavernous sinus Invasion	6 (0.43)	0 (0.00)	4 (0.67)	0.038*
Invasion of Meckel’s cave	8 (0.57)	1 (0.14)	3 (0.50)	0.208
Transtentorial extension	6 (0.43)	1 (0.14)	1 (0.17)	0.337
Clival involvement	9 (0.64)	4 (0.57)	2 (0.33)	0.521
Preoperative hearing loss ^a^	2 (0.14)	2 (0.29)	0 (0.00)	0.513
Preoperative facial numbness/pain ^b^	4 (0.29)	1 (0.14)	2 (0.33)	0.729

GTR, gross total resection; CSF, cerebrospinal fluid. Data are presented as mean ± standard deviation or number (proportion). ^a^ Hearing loss was defined as a pure-tone average threshold greater than 25 dB HL in the affected ear. ^b^ Facial numbness or pain was documented based on patient-reported symptoms and confirmed by preoperative neurological examination. (Definitions for functional outcomes are provided in the Methods Section 2.7). # For the other tumor pathology groups, intergroup statistical comparisons were not performed due to the extremely small number of cases in each group.

**Table 2:** Analysis of the meningioma cohort: baseline and outcome profiles within the location-driven framework (*n* = 20).

Parameter	Kawase approach (*n* = 8)	Retrosigmoid approach (*n* = 7)	Pterional approach (*n* = 5)	P-value
A. Key baseline characteristics				
Age (years)	54.13 ± 1.65	58.86 ± 0.71	58.20 ± 1.24	0.055
Gender (male/female)	3/5	4/3	3/2	0.735
Tumor laterality (left/right)	4/4	3/4	4/1	0.535
Maximum tumor diameter (cm)	2.49 ± 0.30	2.77 ± 0.55	2.48 ± 0.25	0.953
Petrous apex subdivision				<0.001*
Anteromedial	8 (1.00)	0 (0.00)	0 (0.00)	
Posterior	0 (0.00)	7 (1.00)	0 (0.00)	
Superoanterior	0 (0.00)	0 (0.00)	5 (1.00)	
Extension				
Cavernous sinus invasion	5 (0.63)	0 (0.00)	4 (0.80)	0.010*
Invasion of Meckel’s cave	5 (0.63)	1 (0.14)	3 (0.60)	0.139
Transtentorial extension	4 (0.50)	1 (0.14)	1 (0.20)	0.397
Clival involvement	7 (0.88)	4 (0.57)	1 (0.20)	0.054
Preoperative hearing loss ^a^	1 (0.13)	2 (0.29)	0 (0.00)	0.582
Preoperative facial numbness/pain ^b^	3 (0.38)	1 (0.14)	1 (0.20)	0.810
B. Surgery-related outcomes				
Operative time (h)	3.98 ± 0.29	2.72 ± 0.11	2.63 ± 0.15	0.002*
Blood loss (ml)	287.50 ± 12.50	292.86 ± 36.89	230.00 ± 20.00	0.150
GTR	8 (1.00)	6 (0.86)	3 (0.60)	0.150
Trigeminal injury ^c^	0 (0.00)	0 (0.00)	3 (0.60)	0.009*
Facial palsy, HB grade ≥ III	0 (0.00)	1 (0.14)	4 (0.80)	0.004*
Ocular motility deficit	2 (0.25)	0 (0.00)	0 (0.00)	0.311
Motor weakness, MRC grade ≤ 4	0 (0.00)	3 (0.43)	1 (0.20)	0.090
Speech impairment ^d^	0 (0.00)	0 (0.00)	3 (0.60)	0.009*
CSF leak	3 (0.38)	0 (0.00)	0 (0.00)	0.089
consciousness impairment, GCS <15	2 (0.25)	2 (0.29)	0 (0.00)	0.624
Intracranial hemorrhage	0 (0.00)	1 (0.14)	0 (0.00)	0.600
Intracranial infection	6 (0.75)	1 (0.14)	0 (0.00)	0.009*
Hospital stay (days)	48 ± 8	27 ± 3	31 ± 2	0.198

SD, standard deviation; GTR, gross total resection; HB, House-Brackmann; MRC, Medical Research Council; GCS, Glasgow Coma Scale. ^a^ Hearing loss was defined as a pure-tone average threshold greater than 25 dB HL in the affected ear. ^b^ Facial numbness or pain was documented based on patient-reported symptoms and confirmed by preoperative neurological examination. ^c^ Trigeminal sensory hypoesthesia was assessed postoperatively using a standardized sensory examination. ^d^ Speech impairment was defined as newly developed dysarthria or aphasia following surgery. (Definitions for functional outcomes are provided in the Methods Section 2.7).

### 3.2 Clinical outcomes associated with the three surgical approaches

Perioperative clinical outcomes for the three patient groups, stratified by the anatomy-driven surgical approach, are summarized in Table 3.

**Table 3:** Clinical outcomes associated with the three surgical approaches.

Parameter	Kawase approach (*n* = 14)	Retrosigmoid approach (*n* = 7)	Pterional approach (*n* = 6)	*P*-value
Operative time (h)	3.67 ± 0.21	2.72 ± 0.11	2.54 ± 0.15	0.001*
Blood loss (ml)	271.43 ± 11.38	292.86 ± 36.89	233.33 ± 16.67	0.246
Extent of resection				0.055
GTR	14 (1.00)	6 (0.86)	4 (0.67)	
NTR	0 (0.00)	1 (0.14)	2 (0.33)	
New trigeminal hypoesthesia ^a^	1 (0.07)	0 (0.00)	3 (0.50)	0.039*
Facial nerve palsy, HB grade ≥ III	1 (0.07)	1 (0.14)	4 (0.67)	0.017*
Ocular motility deficit	2 (0.14)	0 (0.00)	0 (0.00)	0.721
Motor weakness, MRC grade ≤ 4	2 (0.14)	3 (0.43)	1 (0.17)	0.406
Speech impairment ^b^	1 (0.07)	0 (0.00)	3 (0.50)	0.039*
CSF leak	4 (0.29)	0 (0.00)	0 (0.00)	0.142
Consciousness impairment, GCS <15	3 (0.21)	2 (0.29)	0 (0.00)	0.550
Intracranial hemorrhage	1 (0.07)	1 (0.14)	0 (0.00)	1.000
Intracranial infection	9 (0.64)	1 (0.14)	0 (0.00)	0.009*
Hospital stay (days)#	40 ± 5	27 ± 3	29 ± 2	0.486

GTR, gross total resection; NTR, near-total resection; CSF, cerebrospinal fluid; HB, House-Brackmann grading system; MRC, Medical Research Council. GCS, Glasgow Coma Scale. Data are presented as mean ± standard deviation or number (proportion). ^a^ Trigeminal sensory hypoesthesia was assessed postoperatively using a standardized sensory examination. ^b^ Speech impairment was defined as newly developed dysarthria or aphasia following surgery. (Definitions for functional outcomes are provided in the Methods section 2.7). #: Hospital stay is defined as the total duration from admission to discharge, covering the entire perioperative period (preoperative assessment, surgery, and postoperative recovery with complication management, if any).

#### 3.2.1 Kawase approach group (patients with anteromedial petrous apex lesions)

For lesions located in the anteromedial petrous apex (*n* = 14), the Kawase approach achieved a radiographic gross total resection rate of 100% (14/14). The mean operative time in this group was 3.67 ± 0.21 h, the longest in this series. The mean intraoperative blood loss was 271.43 ± 11.38 mL. The postoperative intracranial infection rate was relatively high (9/14, 64.3%), and all cerebrospinal fluid leak cases occurred in this group (4/14, 28.6%). The incidence of new cranial nerve deficits was relatively low, with one case of trigeminal nerve injury and one case of facial nerve palsy.

#### 3.2.2 Retrosigmoid approach group (patients with posterior petrous apex lesions)

For lesions located in the posterior petrous apex (*n* = 7), the endoscope-assisted retrosigmoid approach achieved an 85.7% (6/7) gross total resection rate. The mean operative time in this group was 2.72 ± 0.11 h, with an average blood loss of 292.86 ± 36.89 mL. The complication profile was relatively balanced, with no single type of complication showing a concentrated high incidence. No cases developed new trigeminal nerve injury, speech impairment, or cerebrospinal fluid leak.

#### 3.2.3 Pterional approach group (patients with superoanterior petrous apex lesions)

For lesions located in the superoanterior petrous apex (*n* = 6), the pterional approach achieved a gross total resection rate of 66.7% (4/6). The mean operative time in this group was 2.54 ± 0.15 h, with an average blood loss of 233.33 ± 16.67 mL. However, the incidence of new postoperative cranial nerve deficits was notably higher: trigeminal nerve injury occurred in 50% (3/6) of cases, facial nerve palsy in 67% (4/6), and speech impairment in 50% (3/6). No cases of intracranial infection or cerebrospinal fluid leak were observed.

In summary, distinct perioperative clinical outcomes were observed among patients undergoing the three different surgical approaches. The distribution of factors such as operative duration, cranial nerve dysfunction, and intracranial infection was notably uneven. These differences may be attributable to the characteristics of the surgical approaches themselves and/or to the inherent anatomy of the lesions. These aspects will be explored in greater depth in the Discussion section.

### 3.3 Outcome profiles by pathological type within the location-driven framework

Given the distinct growth patterns and invasiveness of different pathological types toward surrounding structures, we further examined the surgical outcome characteristics of each pathology under the principle that “location determines approach.” This allows us to appreciate how pathological type interacts with specific “location-approach” pairings, thereby influencing therapeutic outcomes (Table 4). In parallel, we focused on analyzing the largest subgroup—meningiomas (Table 2)—to provide clearer descriptive insights.

**Table 4:** Case distribution and outcomes by pathological type within the location-driven framework.

Pathological type	Total cases (n)	Cases by approach (n)	GTR (n)	Complications
Meningioma	20	K: 8	K: 8 (1.00)	K: Trigeminal injury (*n* = 1), Ocular motility deficit (*n* = 2), CSF leak (*n* = 3), consciousness impairment (*n* = 1), intracranial infection (*n* = 6).
		R: 7	R: 6 (0.86)	R: Facial palsy (*n* = 1), Motor weakness (*n* = 2), Consciousness impairment (*n* = 2), Intracranial hemorrhage (*n* = 1), Intracranial infection (*n* = 1).
		P: 5	P: 3 (0.60)	P: Trigeminal nerve injury (*n* = 3), Facial palsy (*n* = 4), Motor weakness (*n* = 1), Speech impairment (*n* = 3).
Schwannoma	3	K: 2	K: 2 (1.00)	K: Facial palsy (*n* = 1), Motor weakness (*n* = 1), Intracranial infection (*n* = 2).
		P: 1	P: 0 (0.00)	P: –
Cyst	2	K: 2	K: 2 (1.00)	K: CSF leak (*n* = 1), Intracranial infection (*n* = 1).
Cholesteatoma	1	K: 1	K: 1 (1.00)	K: –
Chordoma	1	K: 1	K: 1 (1.00)	K: Motor weakness (*n* = 1), Speech impairment (*n* = 1), Consciousness impairment (*n* = 1), Intracranial hemorrhage (*n* = 1).

K, Kawase approach; R, Retrosigmoid approach; P, Pterional approach.

#### 3.3.1 Meningioma

Among meningioma patients, the following patterns were observed: For meningiomas located in the anteromedial petrous apex, the Kawase approach achieved a high gross total resection rate (8/8), though it was accompanied by a considerable rate of postoperative complications such as intracranial infection. For meningiomas situated in the superoanterior petrous apex, the pterional approach was associated with cranial nerve dysfunction, including trigeminal nerve injury and facial nerve palsy. For meningiomas located in the posterior petrous apex, the endoscope-assisted retrosigmoid approach exhibited a relatively balanced postoperative complication profile and favorable resection outcomes. These findings indicate that, even within the single pathological entity of meningioma, the anatomical location of the lesion—which dictates the choice of surgical approach—remains the most critical factor determining clinical results.

#### 3.3.2 Other pathological types

For other pathological types, due to the limited sample size, the results are presented as descriptive only. Among the 3 patients with schwannoma, 2 underwent the Kawase approach, both achieving GTR; complications included facial palsy (*n* = 1), motor weakness (*n* = 1), and intracranial infection (*n* = 2). One patient underwent the pterional approach, did not achieve GTR, and had no significant complications. Notably, chordomas and complex cysts in this series were also primarily managed via the Kawase approach to address their intricate anatomy within the anteromedial petrous apex, and GTR was achieved in all cases. Complications in the cyst group included CSF leak (*n* = 1) and intracranial infection (*n* = 1). Complications in the chordoma case included motor weakness (*n* = 1), speech impairment (*n* = 1), consciousness disturbance (*n* = 1), and intracranial hemorrhage (*n* = 1). The single patient with cholesteatoma underwent the Kawase approach, achieved GTR, and had no significant complications.

### 3.4 Follow-up outcomes

Among the 27 patients, postoperative follow-up data were available for 21 patients (Kawase approach group: *n* = 11; retrosigmoid approach group: *n* = 5; pterional approach group: *n* = 5). The median follow-up time varied across groups (Kawase: 16 [3–36] months; retrosigmoid: 10 [3–18] months; pterional: 12 [6–24] months) (Table 5).

**Table 5:** Long-term follow-up outcomes associated with the surgical approaches.

Parameter	Kawase approach (*n* = 14)	Retrosigmoid approach (*n* = 7)	Pterional approach (*n* = 6)
Patients with follow-up (n)	11	5	5
Median follow-up time (months)	16 (3–36)	10 (3–18)	12 (6–24)
Tumor recurrence/progression (n)	0	0	1
Trigeminal nerve function recovery/improvement (n/N)*^a^	1/1	–	1/2
Facial nerve function recovery/improvement (n/N)*^b^	1/1	1/1	2/3
Motor recovery/improvement (n/N)*^c^	2/2	2/2	1/1
Speech Recovery/improvement (n/N)*^d^	1/1	0/0	2/2
CSF leak recurrence (n/N)*	0/4	–	–
Intracranial infection recurrence (n/N)*	0/7	0/1	–

*n/N: For complication outcome measures, *N* denotes the number of patients who developed the complication postoperatively and had follow-up data. The ‘n’ represents cases with a favorable outcome: for functional deficits (e.g., nerve palsy), it indicates improvement or resolution; for recurrence-type outcomes (e.g., CSF leak, infection), it indicates cases without recurrence. ^a^ Improvement in trigeminal nerve function was defined as a discernible recovery—either subjective or objective—in light touch or pinprick sensation within facial areas of postoperative sensory deficit, when compared to the neurological status at discharge. ^b^ Defined as improvement of ≥1 grade on the House-Brackmann scale. ^c^ Defined as improvement of ≥1 grade on the MRC muscle strength scale. ^d^ Defined as improvement or resolution of postoperative dysarthria/aphasia on follow-up evaluation. (Definitions for functional outcomes are provided in the Methods Section 2.7).

Regarding the long-term outcomes of complications, the following were observed: For facial nerve function, among the 3 patients in the pterional group who developed this complication and were followed up, 2 showed improvement. In the Kawase and retrosigmoid groups, 1 patient each developed and was followed for this complication, and both showed improvement. For trigeminal nerve function, 1 patient in the Kawase group who developed the complication and was followed up showed improvement. In the pterional group, only 1 of the 2 patients with this injury showed improvement. Other complications, such as motor weakness and speech impairment, showed improvement in all cases. None of the 4 CSF leak cases or the 7 intracranial infection cases that occurred and were followed in the Kawase group recurred during the follow-up period.

Regarding tumor progression and recurrence, imaging progression was observed in 1 patient in the pterional group (pathology: meningioma) during follow-up. No definite recurrence was observed in the other groups within the available follow-up time.

## 4 Discussion

The anatomy of the petrous apex is highly complex, containing critical structures such as the cavernous sinus, various cranial nerve foramina, Meckel’s cave, the trigeminal nerve, and Dorello’s canal with the abducens nerve. During surgical procedures, it is essential to maximize the exposure of the surgical field to facilitate tumor resection, while simultaneously ensuring the protection of surrounding neurovascular structures. Selecting the appropriate surgical approach is crucial for balancing maximal tumor resection and minimizing postoperative complications. The aim of this study was to evaluate the treatment outcomes of three approaches—the Kawase, endoscope-assisted retrosigmoid, and pterional approaches—for petrous apex lesions, all performed by the same team at a single center. It must be emphasized, however, that this investigation was conducted within the context of real-world clinical decision-making, in which the choice of approach is primarily determined by the specific anatomical location of the lesion. Consequently, the outcome differences observed among the groups reflect the comprehensive risk profile inherent to each “specific approach–specific anatomical region” pairing, rather than a direct comparison of technical superiority or inferiority. Nonetheless, by controlling for the surgical team and center, and by ensuring comparability in baseline demographic characteristics (age, sex), tumor laterality, maximum tumor diameter, and preoperative functional status, the observed outcomes can be meaningfully linked to the technical features of each surgical approach and their respective capacities to address particular anatomical challenges.

### 4.1 The Kawase approach

Accurate identification of Kawase’s triangle (the posteromedial middle fossa triangle) is crucial for the Kawase approach. Its boundaries are defined medially by the trigeminal nerve (V3 segment) and the greater superficial petrosal nerve (GSPN), laterally by the arcuate eminence and the superior semicircular canal, inferiorly by the petrous segment of the internal carotid artery, and superiorly by the trigeminal ganglion (Gasserian ganglion). Kawase’s triangle represents a safe zone for bone drilling (10–12).

Removing the bone within Kawase’s triangle using a drill and craniotome creates a direct communication between the middle and posterior cranial fossae. This corridor not only provides ample room for instrument manipulation but also yields extensive exposure. It facilitates access to lesions located in the anteromedial petrous apex, the petrous segment of the internal carotid artery, the mid to upper clivus, the ventrolateral brainstem cisterns, as well as meningiomas involving both supra- and infratentorial compartments and tumors situated anteromedial to the superior cerebellar surface.

A key advantage of this approach lies in its facilitation of exposure and protection of critical cranial nerves such as the trigeminal and facial nerves. During the procedure, it allows for the full exposure of these nerves along their relevant courses. This enables clear identification and the systematic release of any tumor-induced compression, contributing to more radical resection (13–15).

The Kawase approach group exhibited a high rate of intracranial infection (64.3%, *p* < 0.05). This postoperative complication may be attributed to both the technical demands of the approach—including its complexity, longer operative time, and opening of pneumatized petrous air cells—and the characteristics of the lesions themselves: all patients in this group had lesions in the anteromedial petrous apex with frequent transtentorial extension, necessitating more extensive bone drilling, which likely increased the risk of infection. Consequently, this elevated infection rate should be regarded as an inherent risk associated with employing the Kawase approach for complex anteromedial petrous apex lesions. Furthermore, cerebrospinal fluid (CSF) leaks occurred exclusively in the Kawase group (28.6%), which is directly related to the need for meticulous dural closure after opening petrous air cells to prevent fistula formation (12, 16). Although the incidence of postoperative infection was high, no recurrence was observed during follow-up after appropriate management, indicating that such complications are controllable with active treatment.

### 4.2 The retrosigmoid approach

The retrosigmoid approach combined with neuroendoscopy provides considerable visual access to the cerebellopontine angle and its surrounding neurovascular structures. It offers excellent exposure of the posterior cranial fossa, cerebellopontine angle, internal auditory canal, ventral brainstem, superior petrosal vein, and cranial nerves V through XII. Furthermore, neuroendoscopy facilitates clear visualization of the relationship between the tumor and adjacent vessels, effectively expanding the surgical field of view (17, 18).

For patients with posterior petrous apex lesions in this study, this technique allows for better protection of the facial, vestibulocochlear, and trigeminal nerves, helps reduce vertigo caused by vestibular apparatus injury, and minimizes overall surgical trauma. While the retrosigmoid approach combined with neuroendoscopy is highly effective for tumors located posteromedially and those extending into the cerebellopontine angle, its utility is limited for tumors situated in the anterior petrous apex, closely adherent to the clivus or the posterior portion of the cavernous sinus. In such cases, the line of sight via the retrosigmoid approach becomes obstructed, significantly increasing the technical difficulty of the procedure (19, 20). This further confirms that selecting the appropriate surgical approach based on the core anatomical location of the lesion is fundamental to achieving optimal exposure and minimizing postoperative complications.

### 4.3 The pterional approach

The pterional approach is widely utilized in lateral skull base surgery. It provides extensive exposure of the sylvian fissure, sellar region, anterior clinoid process, optic nerve, internal carotid artery, and its bifurcation. This approach is particularly suitable for lesions located in the superoanterior petrous apex with extension to the posterior cavernous sinus and parasellar region (21, 22).

In this study, the pterional approach group exhibited a significantly higher incidence of trigeminal nerve injury, facial nerve injury, and speech impairment (*p* < 0.05). However, all lesions in this group were located in the superoanterior petrous apex and frequently involved the cavernous sinus region. These anatomical areas are intrinsically associated with relevant cranial nerves; for example, cavernous sinus pathology often affects branches of the trigeminal nerve. Therefore, the observed high rate of postoperative neurological deficits may be attributable to the inherent nature of the lesions themselves, the challenging anatomy of the region, and/or the combined effects of the exposure provided by the pterional approach and intraoperative manipulation, including retraction of the temporal lobe and cranial nerves. This suggests that for lesions in this specific location, the risk of cranial nerve injury may be inherently elevated regardless of the surgical approach chosen. Under such high-risk anatomical conditions, the pterional approach did not demonstrate a clearly superior protective effect (23, 24). Furthermore, follow-up data from patients in this group showed that some who experienced nerve injuries exhibited functional improvement over time, suggesting that these deficits may be partially related to reversible factors such as surgical retraction.

### 4.4 Interaction between pathological type and surgical outcomes within the location-based framework

This study encompasses common petrous apex pathologies, including meningiomas, schwannomas, cholesteatomas, cysts, and chordomas. It must be re-emphasized that surgical approach selection in this study consistently adhered to the principle of “anatomical location first.” Even within the largest meningioma subgroup, the lesion locations differed across the corresponding approach groups. Therefore, the pathological subgroup analysis conducted herein aimed to examine how distinct pathological characteristics interact with the technical specifics of a given approach—after the lesion location had predetermined the fundamental surgical corridor—thereby influencing the ultimate extent of resection and the profile of complications.

#### 4.4.1 Meningioma

This type of tumor frequently involves the petrous apex, petroclival region, and cerebellopontine angle. Meningiomas are typically hypervascular and adherent to the dura (3). For meningiomas located in the anteromedial petrous apex, the Kawase approach provides an ideal corridor for simultaneously addressing lesions in both the middle and posterior cranial fossae, achieving a high gross total resection rate (8/8). However, the extensive petrous bone drilling and longer operative time required by this approach may be associated with a higher risk of postoperative infection. For meningiomas situated in the posterior petrous apex, the endoscope-assisted retrosigmoid approach offers excellent direct visualization, allowing for a high resection rate (6/7) while preserving neurovascular structures, and results in a relatively balanced complication profile. For meningiomas located in the superoanterior petrous apex, the pterional approach allows direct access to the parasellar region, but its working space is limited when dealing with lesions that frequently involve the cavernous sinus, potentially leading to a higher risk of cranial nerve injury (3 cases of trigeminal nerve injury, 4 cases of facial nerve palsy). This suggests that the pathological characteristics of meningiomas may amplify the inherent anatomical challenges.

#### 4.4.2 Schwannoma

These tumors typically originate from the facial or vestibulocochlear nerve. Therefore, in addition to tumor resection, the primary surgical goals are the preservation of facial nerve function and hearing (25, 26). Of the three schwannomas in this cohort, two were located in the anteromedial petrous apex and were therefore approached via the Kawase route. This approach facilitates early extradural identification of the trigeminal nerve at Meckel’s cave, providing favorable conditions for achieving gross total resection (2/2) while preserving neural anatomy. The remaining tumor, situated in the superoanterior petrous apex, was addressed using the pterional approach, resulting in subtotal resection without new postoperative neurological deficits. This preliminary observation suggests that for neurogenic tumors, irrespective of the specific approach, a surgical design that enables early identification and protection of the involved nerve is crucial for functional outcome.

Both cholesteatoma and chordoma are characterized by aggressive growth; therefore, complete resection is crucial to minimize recurrence (27, 28). In this study, the cysts (2 cases), cholesteatoma (1 case), and chordoma (1 case) were all located in the anteromedial petrous apex and were therefore surgically addressed via the Kawase approach. The multi-angular, wide-field exposure afforded by the Kawase approach in this region aligns well with the surgical objectives for these pathologies: decompression and nerve preservation for cysts, and pursuit of gross total resection for aggressive lesions. All cases achieved radiographic gross total resection, supporting the view that for lesions in this region—irrespective of pathology—selecting an approach that provides adequate bony resection and surgical exposure constitutes a common surgical goal.

Therefore, the specific anatomical location of the lesion within the petrous apex (anteromedial, posterior, or superoanterior) remains the primary determinant in selecting the surgical approach. Our subgroup analysis of meningiomas further validates this principle, demonstrating that even within a single pathological entity, the distinct complication profile associated with each surgical approach persists.

### 4.5 Analysis of hospital stay duration

In this study, the three “location–approach” pairings exhibited distinct patterns of hospital stay duration: 40 days in the Kawase group (anteromedial petrous apex), 27 days in the retrosigmoid group (posterior petrous apex), and 29 days in the pterional group (superoanterior petrous apex). The variation in length of stay—particularly the prolonged hospitalization in the Kawase group—serves as an important descriptive indicator reflecting the overall treatment course. This difference is primarily attributable to the following factors.

First, it is closely related to the management and care of complications. The Kawase group had the highest rate of intracranial infection (64.3%) and accounted for all cerebrospinal fluid (CSF) leak cases (28.6%). Antibiotic therapy and continuous lumbar drainage are required to treat these complications significantly prolonged hospitalization. Second, the anatomical complexity of the lesions dictates postoperative monitoring needs. Anteromedial petrous apex lesions are deeply situated, adjacent to the brainstem and critical neurovascular structures, typically necessitating closer neurological monitoring and a longer recovery process postoperatively. In contrast, although the pterional group had a higher incidence of new cranial nerve deficits, such functional impairments often recovered during outpatient follow-up and had less impact on acute-phase hospital stay. The endoscope-assisted retrosigmoid group, with its more balanced complication profile and minimal tissue trauma, had the shortest hospital stay. Therefore, hospitalization duration also reflects an important clinical characteristic of the inherent risks, complication spectrum, and recovery process associated with surgery in specific anatomical regions, providing valuable reference for preoperative assessment.

### 4.6 Role and future prospects of neuroendoscopy

Based on our findings, the retrosigmoid approach combined with neuroendoscopy provides significantly enhanced visualization, offering a substantially larger field of view and greater depth of field compared to the microscope alone (29, 30). With endoscopic assistance, surgical maneuvers become more precise, which significantly reduces the risk of injury to vessels, nerves, and the internal auditory canal. This technique also enables more accurate and complete tumor resection, thereby lowering the likelihood of recurrence. Neuroendoscopy is minimally invasive, requiring only a small incision and utilizing natural anatomical corridors, which also minimizes the need for brain retraction (31–33).

In the future, integrating neuroendoscopy with the Kawase approach could address the anatomical limitations inaccessible via the endoscopic retrosigmoid route. After drilling Kawase’s triangle, the use of a neuroendoscope can expand the exposure of the petrous apex, petroclival region, and the ventrolateral brainstem. Further extensive drilling of the petrous apex will correspondingly increase the exposed area. This hybrid strategy not only maximizes the surgical field and reduces technical difficulty but also maintains a minimal trauma profile for the patient. The combination of the Kawase approach and neuroendoscopy holds the potential to optimize both the gross total resection rate and the control of postoperative complications.

### 4.7 Hybrid surgical strategy within the anatomy-based framework

Our study provides a clear delineation of the respective advantages and limitations of the three surgical approaches. To maximize the rate of gross total resection and minimize patient complications, a hybrid strategy, partially combining elements of these approaches, can be employed. This is not a simple superposition of techniques, but rather a sequential and staged process tailored to the individual patient’s anatomy and the specific phase of the procedure, allowing for controlled exposure and resection of the tumor (34).

Our work is founded upon a hierarchical decision-making framework based on the principle of “anatomical location first.” A hybrid approach can be strategically planned as follows: For tumors involving the anteromedial petrous apex and both the middle and posterior cranial fossae, the Kawase approach combined with neuroendoscopy is recommended. For tumors located in the posterior petrous apex, posterior fossa, and cerebellopontine angle in patients with pre-existing hearing loss, the retrosigmoid approach combined with neuroendoscopy is an effective option. For large tumors extensively involving the petrous apex with simultaneous extension into the parasellar region, middle fossa, and posterior fossa, a combination of the pterional and Kawase approaches may be utilized.

## 5 Limitations and future directions

Although this study provides a detailed real-world clinical description of outcomes for three mainstream surgical approaches to petrous apex lesions, several important limitations must be acknowledged. First, the study has a retrospective, non-randomized, and “anatomy-driven” design. The selection of surgical approach is inherently tied to the core anatomical location of the lesion. This results in systematic and intrinsic differences between the comparison groups regarding the most critical prognostic factor. Consequently, while the distinctive complication profiles we observed likely reflect the combined influence of both the technical characteristics of the surgical approaches themselves and the inherent anatomical complexity of the lesions they were used to address, this study describes the outcome spectrum associated with specific “anatomical location–surgical approach” pairings in clinical practice, rather than aiming to provide confirmatory evidence of the isolated effect of a surgical technique. Second, due to the rarity of petrous apex lesions, the number of cases included in this study is limited, which may result in insufficient statistical power. Although a subgroup analysis based on pathological type was performed, some subgroups (e.g., cholesteatoma, chordoma) contained only single cases; therefore, these findings are descriptive and exploratory, requiring validation through studies with larger sample sizes. Third, as a single-center retrospective study, data collection is subject to inherent constraints. Despite using a standardized multidisciplinary discussion to determine the surgical approach and a consistent surgical team to minimize operator bias, we cannot completely rule out unmeasured confounding factors or potential selection bias. Fourth, follow-up duration and assessment content varied among patients, which may affect the accurate evaluation of long-term tumor control and functional outcomes.

Despite these limitations, this study nevertheless holds significant value. It is the first to systematically delineate the spectrum of outcomes associated with three commonly used approaches—each applied according to mainstream clinical rationale within its respective anatomical indications—when performed by the same surgical team. This provides an important reference for preoperative communication and surgical planning.

Future studies should aim to validate these findings through prospective, multicenter collaboration to accumulate larger and more robust datasets. In such large cohorts, applying propensity score matching or multivariable regression analysis would help statistically adjust for baseline anatomical differences, thereby enabling a more precise delineation of the independent contribution of each surgical approach. Furthermore, integrating neuroendoscopy with hybrid surgical approaches and advanced intraoperative technologies—such as neuronavigation, intraoperative neuromonitoring, and augmented reality—holds promise for further improving the safety and efficacy of these procedures.

## 6 Conclusion

Through an analysis of 27 patients, this study systematically delineates the comprehensive outcomes of treating petrous apex lesions using the Kawase approach, the endoscope-assisted retrosigmoid approach, and the pterional approach within the clinical practice of prioritizing “anatomical location as the primary selection criterion.” Our findings reveal a characteristic spectrum of outcomes closely tied to the anatomical indications of each approach: for anteromedial petrous apex lesions, the Kawase approach provides excellent exposure to achieve a high gross-total resection rate, yet it is associated with longer operative times and a higher risk of postoperative intracranial infection in this patient group. For posterior petrous apex lesions, the endoscope-assisted retrosigmoid approach offers ideal exposure with minimal tissue trauma and a relatively balanced complication profile. For superoanterior petrous apex lesions, the pterional approach enables direct access, but patients in this group exhibited a higher risk of cranial nerve dysfunction (trigeminal and facial nerves) and speech impairment. Therefore, after selecting the primary approach based on lesion location, surgeons should fully recognize and proactively guard against the characteristic perioperative risks associated with that approach and its corresponding anatomical region. In the future, integrating ancillary techniques such as neuroendoscopy with these classic approaches may further optimize surgical outcomes in complex cases.

## Data Availability

The original contributions presented in the study are included in the article/Supplementary material, further inquiries can be directed to the corresponding author.
